# Current use of mechanical bowel preparation and oral antibiotics before elective colorectal resection in Australia and New Zealand

**DOI:** 10.1111/ans.70052

**Published:** 2025-03-05

**Authors:** Claudia Paterson, Andrew Hill, Parry Singh

**Affiliations:** ^1^ Department of Surgery Te Whatu Ora – Counties Manukau, University of Auckland Auckland New Zealand

## Introduction

Use of mechanical bowel preparation (MBP) and oral antibiotics (OABs) is contentious in colorectal surgery (CRS). MBP + OABs were commonplace in the 1970s, but the following decades saw a reduction in MBP, and an increase in the use of intravenous antibiotics (IVABs).^1^ Over the 2010s, large‐scale retrospective analyses demonstrated an association between MBP + OABs and reduced surgical site infection (SSI) and anastomotic leak (AL).^2^, ^3^, ^4^ This argument has been strengthened by meta‐analyses, resulting in the recent resurgence of MBP + OABs.^5^, ^6^

Current American guidelines recommend MBP + OABs before CRS.^7^ Contrastingly, current Australian guidelines do not recommend MBP before colon surgery,^8^ and do not mention rectal surgery or OABs. This is similar to the current Enhanced Recovery After Surgery guidelines, but also comments MBP may be used before rectal surgery.^9^

The intestinal microbiota has gathered interest for its role in infectious complications.^10^ MBP + OABs indiscriminately reduce bacterial load, which inadvertently harms commensal bacteria.^11^ Probiotics are well known among the public, and their role in reducing postoperative infections is of interest.^12^

Our primary aim was to establish current use of MBP + OABs before CRS in Australia and New Zealand (ANZ). ANZ colorectal surgeons were last surveyed in 2019.^13^ Our secondary aim was to assess current recommendations regarding probiotics.

## Methods

Approval was obtained from the Auckland Health Research Ethics Committee (ref: AH27488). This anonymous and voluntary survey was distributed to Colorectal Surgical Society of ANZ members via email on 11 June 2024. Unique codes were provided, and informed consent was taken on survey submission. Another email was sent on 24 June 2024, with the last survey completed on 22 August 2024. Data were stored on REDCap. Summary statistics and graphical displays were formulated using R version 4.4.1. Data were presented to one decimal place, except when comparisons are performed to previous surveys, where whole numbers were used for consistency. Routine and selective use of MBP + OABs were combined for comparison with previous surveys.

## Results

### Demographics

**Fig. 1:**
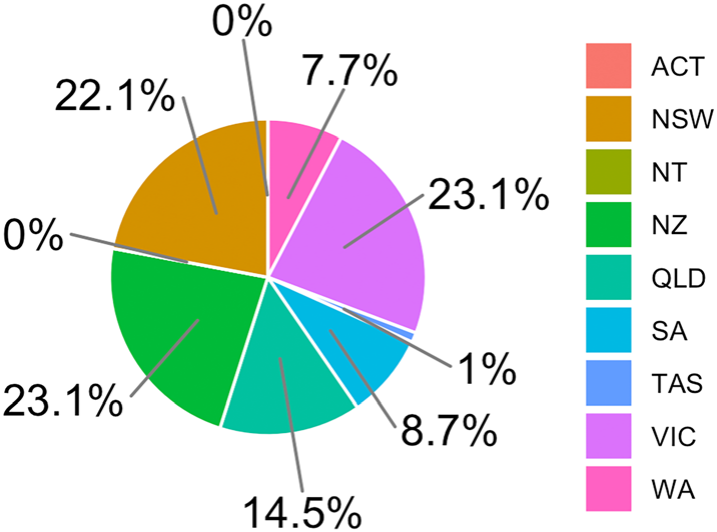
Location distribution among survey respondents

104/356 (29.2%) members responded. The median age was 48 years old (IQR 41–56). 81.7% identified as male, 17.3% as female and 1.0% as other. The median annual number of colon resections was 50 (IQR 30–80), and rectal resections, 15 (IQR 10–20). 83.7% worked at a tertiary centre. 23.1% were from New Zealand, and 76.9% from Australia (Fig. 1).

### MBP

MBP was used routinely in 44.3% of right colon, 70.2% of left colon, and 85.6% of rectal resections (Table 1). Of those who used MBP, the three most selected types were oral picosulfate (63 respondents), oral polyethylene glycol (PEG) (46 respondents), and rectal sodium phosphate (26 respondents). Oral PEG + rectal sodium phosphate was the most common combination (nine respondents).

**Table 1:** MBP use in colon and rectal resections

	Right colon	Left colon	Rectal
Routine	44.3%	70.2%	85.6%
Selective	11.5%	11.5%	12.5%
Do not use	44.2%	18.3%	1.9%

### OABs

OABs were used routinely in 21.2% of right colon and 26.0% of left colon and rectal resections (Table 2). Of those who used OABs, the three most selected types were metronidazole (21 respondents), neomycin (12 respondents), and ciprofloxacin (eight respondents). Erythromycin, co‐trimoxazole, clindamycin, and cefalexin were selected at least once. Metronidazole + neomycin was the most common combination (seven respondents). Twenty‐six respondents used OABs 1 day before surgery, and two respondents used OABs for 2 days before surgery.

**Table 2:** OABs use in colon and rectal resections

	Right colon	Left colon	Rectal
Routine	21.2%	26.0%	26.0%
Selective	2.9%	1.0%	1.0%
Do not use	75.9%	73.0%	73.0%

### MBP + OABs

**Fig. 2:**
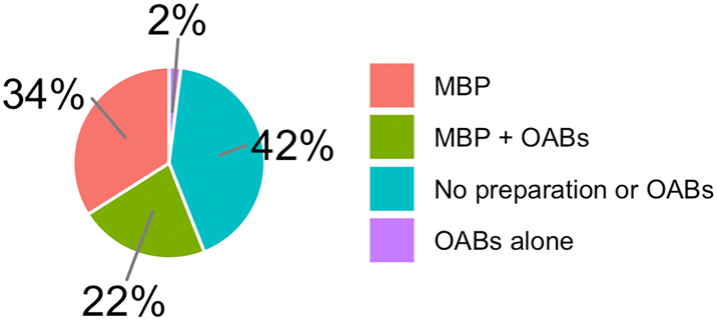
Use of MBP + OABs in right‐sided colon resections

**Fig. 3:**
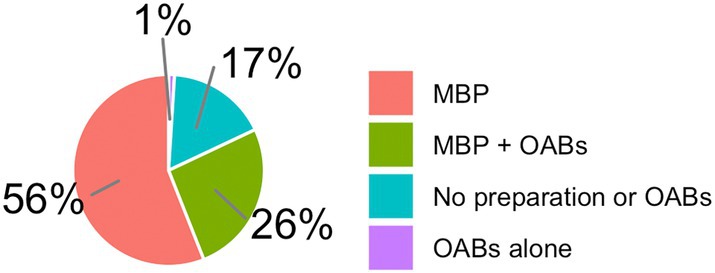
Use of MBP + OABs in left‐sided colon resections

**Fig. 4:**
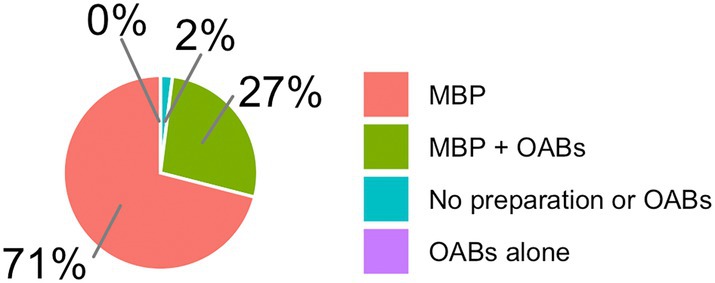
Use of MBP + OABs in rectal resections

MBP + OABs was used by 22%, MBP by 34%, OABs alone by 2%, and no preparation or OABs by 42% in right colon resections (Fig. 2). For left colon resections, MBP + OABs was used by 26%, MBP in 56%, OABs alone in 1%, and no preparation or OABs in 17% (Fig. 3). For rectal resections, MBP + OABs was used by 27%, MBP by 71%, OABs alone by 0%, and no preparation or OABs by 2% (Fig. 4).

### Changes over time

MBP + OABs increased from 10% in colon resections in 2019 to 22% in right colon and 26% in left‐sided colon resections in 2024 (Table 3). For rectal resections, MBPs + OABs increased from 14% in 2019 to 27% in 2024 (Table 4).

**Table 3:** Changes in MBP + OABs use in colon resections over the past 5 years.

	Colon (2019)^12^	Right colon (2024)	Left colon (2024)
MBP + OABs	10%	22%	26%
MBP	45%	34%	56%
OABs alone	0%	2%	1%
No preparation or OABs	45%	42%	17%

**Table 4:** Changes in MBP + OABs use in rectal resections over the past 5 years

	Rectal (2019)^12^	Rectal (2024)
MBP + OABs	14%	27%
MBP	81%	71%
OABs alone	0%	0%
No preparation or OABs	5%	2%

### Changes to practice

Of those who have changed their MBP practice, 24 reported using more and 16 reported using less. Of those who have changed their OABs practice, all 27 reported using more. Thirty‐eight reported that new evidence had the biggest change to practice (Table 5).

**Table 5:** Changes to practice over the past decade

Question	No	Yes	
Has your practice regarding MBP changed in the past 10 years?	64	40	24 using more
			16 using less
Has your practice regarding OABs changed in the past 10 years?	77	27	27 using more
			0 using less

	Factors		Respondents
Which factors have had the biggest change to practice?	New evidence being published		38
	Change in personal preference		9
	Departmental guidelines		2
	Intra‐corporeal anastomosis		1

### Operative factors

Planning for a stoma made 51 respondents more likely to use MBP, 51 reported no effect, and two were less likely (Table 6). Most indicated that planning for a stoma would not affect their decision to use OABs. Most reported that a planned minimally invasive approach would not change their use of MBP or OABs.

**Table 6:** How different operative factors would influence likelihood of using MBP/OABs.

Scenario	More likely	No change	Less likely
How would preoperative planning for a stoma change your decision to use MBP?	51	51	2
How would preoperative planning for a stoma change your decision to use OABs?	6	96	2
How would a planned minimally invasive approach change your decision to use MBP?	19	84	1
How would a planned minimally invasive approach change your decision to use OABs?	3	101	0

### Probiotics

15 (14.4%) recommended probiotics. Of those who recommended types of probiotics, seven recommended non‐specific brands, two recommended Yakult, one recommended Inner Health Plus, and one recommended Meta Align.

## Discussion

In ANZ, since 2019, use of MBP + OABs has more than doubled before elective colon resections, and almost doubled before elective rectal resections. However, MBP + OABs are not used by most members. Use of OABs alone remains uncommon. 14.4% reported recommending probiotics, but most did not recommend a specific type.

The most common OAB combination was metronidazole and neomycin, but this was not unanimous. Heterogeneity was likely due to limited availability of neomycin, resulting in selection of alternative antibiotics. Neomycin has the most international evidence, but is not available in New Zealand. Neomycin has recently been made more widely available in Australia, allowing for before/after retrospective cohort studies. One group demonstrated that MBP + neomycin and metronidazole reduced AL compared to MBP,^14^ and another group showed that MBP + neomycin and metronidazole was associated with decreased superficial SSI compared to MBP or no preparation.^15^ The upcoming Colorectal Anti‐Bacterial Eradication (CABE) trial is investigating whether OABs (neomycin and metronidazole) can reduce AL and SSI compared to placebo (with or without MBP). Results will be of significant interest and potentially improve access to neomycin across ANZ.^16^

We anticipate that use of MBP + OABs will continually rise before elective CRS, supported by ongoing evidence on infectious complications. Collaborative efforts with local infectious disease specialists are essential to ensure that the selected OABs remain effective. Data on adverse effects, such as electrolyte disturbances and *Clostridium difficile* colitis, should be published, to better identify patients at risk of these complications.

We speculate that the intestinal microbiota will play a growing role in our understanding of infectious complications. Microbiota‐modulating agents, such as probiotics or faecal microbiota transplantation, may be able to provide patient‐specific interventions.

Our response rate of 29.2% response rate is comparable to other studies,^13^, ^17^, ^18^ with the 2019 survey of the same cohort having a response rate of 31.7%.^13^ 83.7% worked at a tertiary centre, with no respondents from Australian Capital Territory or Northern Territory. Over‐representation from urban/academic hospitals is a potential weakness of this survey.

In conclusion, use of MBP + OABs before CRS has increased in ANZ over the past 5 years. This aligns with current evidence regarding infective complications. However, many do not use MBP + OABs, particularly before right colon resection. Wider availability of appropriate antibiotics with antimicrobial resistance monitoring, and ongoing research into how MBP + OABs affect the intestinal microbiota should inform future practice.

## Funding information

Claudia Paterson is funded by the Health Research Council of New Zealand on a Clinical Research Training Fellowship.

## Author contributions

**Claudia Paterson:** Conceptualization; data curation; formal analysis; investigation; methodology; project administration; writing – original draft; writing – review and editing. **Andrew Hill:** Conceptualization; data curation; formal analysis; investigation; methodology; supervision; writing – original draft; writing – review and editing. **Parry Singh:** Conceptualization; data curation; formal analysis; investigation; methodology; supervision; writing – original draft; writing – review and editing.
